# Effectiveness of Intraoperative Facial Nerve Monitoring in Submandibular Gland Surgery: A Retrospective Study of a Single Institution

**DOI:** 10.3390/diseases13040096

**Published:** 2025-03-26

**Authors:** Maria Giulia Cristofaro, Francesco Ferragina, Giuseppe Tarallo, Angelo Ruggero Sottile, Maria Grazia Ioppolo, Antonella Arrotta, Teresa Chiara De Bartolo, Ida Barca

**Affiliations:** 1 Maxillofacial Surgery Unit, Department of Experimental and Clinical Medicine, Renato Dulbecco Hospital, Magna Graecia University of Catanzaro, Viale Europa, 88100 Catanzaro, Italy; francesco.ferragina92@gmail.com (F.F.); barca.ida@gmail.com (I.B.); 2 Maxillofacial Surgery Operative Unit, Department of Neurosciences, Reproductive and Odontostomatological Sciences, Federico II University of Naples, 80131 Naples, Italy; giuseppe.tarallo92@gmail.com (G.T.); angelo.sottile.1991@gmail.com (A.R.S.); magioppolo@gmail.com (M.G.I.); 3 Department of Medical and Surgical Sciences, Anesthesia and Intensive Care, Renato Dulbecco Hospital, Magna Graecia University of Catanzaro, Viale Europa, 88100 Catanzaro, Italy; antonella.arrotta@hotmail.it; 4 School of Medicine, “Magna Graecia” University, Viale Europa, 88100 Catanzaro, Italy; teresachiara.debartolo@studenti.unicz.it

**Keywords:** benign pathology submandibular gland, marginalis mandibulae branch, postoperative facial nerve paralysis, intraoperative facial nerve monitoring

## Abstract

Background: Intraoperative facial nerve monitoring (IFNM) is becoming increasingly prevalent as an established intraoperative aid in parotid gland surgery. To date, however, there are few scientific studies on the postoperative outcomes of submandibular gland surgery, particularly on the postoperative injury of the marginalis mandibulae branch (MMB) of the facial nerve (FN). This branch represents the most frequent and feared complication of this surgery, with an incidence of 1–7% of cases. Objective: This retrospective study aims to evaluate the incidence of postoperative MMB paralysis in patients undergoing submandibular sialoadenectomy for benign conditions from 2014 to 2023, focusing on the role of IFNM. Materials and Methods: The patients were divided into two groups: the subjects of Group 1 (G1) had undergone submandibular sialoadenectomy after identification and clamped facial vessels, without the aid of IFNM (from 1 January 2014 to 31 December 2018). Conversely, subjects in Group 2 (G2) underwent IFNM procedures (from 1 January 2019 to 31 December 2023). The classification of any FN malfunctions was conducted following the House–Brackmann grading system. A descriptive analysis was performed, and univariate and multivariate logistic regressions were used to examine the impact of IFNM on surgical timing and the association between G2 deficit (vs. G1) corrected for age, sex, and smoking status. Results: The study population comprised a total of 101 patients with a mean age of 55 ± 16 years. The sample population comprised 50 subjects assigned to Group 1 (49.5%, 24 females and 26 males) and 51 subjects assigned to Group 2 (50.5%, 21 males and 30 females). Postoperative paralysis of the MMB occurred in 23 subjects (22.77%), including 12 of G1 (4 had a grade II and 8 grade III dysfunction) and 11 of G2 (8 had a grade II and 3 grade III dysfunction). A six-month evaluation revealed that only five patients in G1, previously diagnosed with grade II dysfunction, exhibited a residual deficit. The mean surgical time for the entire patient cohort was 99 ± 44 min: 110 ± 43 min for Group 1 and 92 ± 42 min for Group 2 (Beta = −19; 95% CI −37 at −0.16; *p*-value = 0.048). Furthermore, a longer operative time was observed in smokers than in non-smokers (*p*-value = 0.008), suggesting that smoking affects the length of surgery (Beta = 0.32; 95% CI −0.08 to −0.55). Discussion and Conclusions: MMB paralysis is one of the most prevalent complications that may arise in submandibular gland surgery. IFNM provides surgeons with a valuable tool for identifying MMB in submandibular sialoadenectomy. The efficacy of IFNM as an aid is contingent upon the expertise of the operating surgeon.

## 1. Introduction

Intraoperative facial nerve monitoring (IFNM) has been extensively studied in parotid gland surgery, proving to be a valuable aid to the surgeon as it reduces the risk of nerve injury and improves postoperative functional outcomes [1,2,3]. Although these benefits are well documented for parotid surgery [4,5,6,7,8], there is a paucity of the scientific literature that has evaluated the impact of IFNM in submandibular gland surgery, in particular on one of the terminal branches of the facial nerve (FN), the marginalis mandibulae branch (MMB) [3,9,10]. The MMB is thin and is characterized by its role in motor innervation of the lower lip and the chin region (marginal parts of the orbicularis oris, depressor anguli oris, transversus menti, depressor labii inferioris, and mentalis muscles). Notably, anastomosis with other branches is not universally present. Its injury causes motor dysfunction and facial asymmetry, with a significant impact on the patient’s quality of life.

MMB lesions are among the most prevalent (29–30% of cases) and feared complications in submandibular triangle surgery, attributable to their superficial course [11,12,13]. The utilization of retractors during surgery is a frequent contributing factor [3,14,15]. Even simple tissue traction can cause temporary damage; soft tissue edema adversely affects the MMB. Already in 1964, De Sousa et al. demonstrated the importance of the contraction of the platysma muscle in the lowering of the labial commissure and the lower lip using electromyography [16]. The platysma muscle is involved together with the depressor anguli oris muscle in the equilibrium between elevation and depression forces acting on the labial commissure. It also contributes to the depressor labii inferioris, which is responsible for the lowering of the lower lip. Both muscles are innervated by the MMB. The contraction of the platysma, depressor anguli oris, and the depressor labii inferioris muscles is responsible for the expression of sadness, disgust, and bitterness. In the case of a forced smile, the contraction also results in the exposure of the upper and lower dental arches.

Sometimes, the impairment sustained by the cervical branch of the FN (which invariably lies posterior to the MMB and whose anatomy is often poorly understood) is mistakenly attributed to the MMB lesion [17,18]. The anastomotic branching between the MMB and the cervical branch of FN was found to occur exclusively posteriorly or superiorly to the submandibular gland. The mandibular branch typically exists behind or over the antero-inferior sub-aspect of the lower pole of the parotid gland, often in conjunction with the cervical branch. The nerves diverged at a distance from the parotid gland, but they always separated as they reached the angle of the mandible. FN’s cervical branch injuries induce asymmetry in these combined movements due to a reduced counterbalancing effect on the elevator commissures labialis on the affected side, which is especially noticeable when smiling, which is often mistaken for a marginal lesion of the MMB. This asymmetry is found in 35–40% of cases of patients undergoing submandibular sialoadenectomy [19]. It is transitory and generally resolves within a month after surgery. By respecting some simple technical points during the approach to the submandibular gland, the FN can be easily spared. In fact, by sparing the posterior part of the muscle in the platysma section, the risk of injury to its cervical branch is reduced to a minimum without in any way hindering the approach to the submandibular space. The most important complication of submandibular gland surgery is due to the MMB injury rather than FN’s cervical branch [20,21].

Many authors recommend identifying the facial artery and vein near the lower border of the mandible and ligating these vessels before pulling them cranially to protect the nerve. However, it is not uncommon for the MMB to run caudally in this region. While the mandibular branches always pass superficial to the anterior facial vein, the relationship of the nerve to the facial artery is extremely variable [22,23,24].

Although the FN identification method may seem like a valid protection strategy, problems may arise for the following reasons: MMB identification is often difficult in patients with abundant subcutaneous fat, many patients have multiple MMBs, and finally the MMB is a thin nerve, so dissection alone may cause paralysis. It may be useful during the removal of the submandibular gland not to proceed with the direct identification of the nerve (avoiding its expected course) but to trace its path with the aid of the IFNM.

The gap in the literature regarding scientific studies that have addressed iatrogenic damage to the FN in submandibular gland surgery suggests the need for further research to determine whether the introduction of IFNM can offer the same level of protection in this context, providing surgeons with additional assistance in safeguarding the nerve. In this retrospective study, the authors evaluated the incidence of postoperative paralysis of the MMB of the FN in patients undergoing submandibular sialoadenectomy for benign diseases, such as benign tumors and sialolithiasis, from 2014 to 2023, with particular attention to the role of IFNM in this context.

## 2. Materials and Methods

This retrospective study includes patients who underwent submandibular sialoadenectomy for benign diseases at the Department of Maxillofacial Surgery of the “Magna Grecia” University of Catanzaro, from 1 January 2014 to 31 December 2023, with and without the use of IFNM.

The research was approved by the Institutional Review Board and by the Ethics Committee of the “Magna Grecia” University of Catanzaro, Italy (protocol number 000146_16). The study was conducted following the ethical standards set out in the Declaration of Helsinki.

### 2.1. Search Strategy

To facilitate a comparative analysis of data from patients undergoing submandibular sialoadenectomy with and without the use of IFNM, patients were categorized into two distinct groups: Group 1 (G1) underwent submandibular sialoadenectomy without IFNM and with identification and clamping of facial vessels; Group 2 (G2) underwent submandibular sialoadenectomy with the use of IFNM and without identification and clamping of facial vessels. Group 1 included subjects who underwent surgery from 1 January 2014 to 31 December 2018. Group 2 included subjects who underwent surgery between 1 January 2019 and 31 December 2023.

The data obtained from the medical records and histological examinations were organized in a database using Microsoft Excel (Version 2017, Redmond, WA, USA). The database encompassed personal data, tobacco and alcohol habits, comorbidities, other interventions performed, histological diagnosis, presence or absence of facial paralysis with involvement of the MMB, and surgical timing. Subsequent clinical and telephonic follow-ups were utilized to ascertain the resolution or persistence of FN dysfunction.

### 2.2. Inclusion and Exclusion Criteria

The inclusion criteria were as follows: patients of both sexes, without age limits; patients who underwent submandibular sialoadenectomy for benign pathology (sialolithiasis, chronic recurrent sialadenitis, benign tumors); the absence of pre-operative FN dysfunction.

The exclusion criteria were as follows: patients with incomplete clinical and instrumental documentation, patients who had already been surgically treated in the same region for another pathology, and patients who were not cooperating.

To minimize selection bias, the study’s participants were restricted to patients who were treated by two surgeons with comparable levels of expertise (MGC and IB) and the choice of device use was random.

### 2.3. Data Extraction and Quality Assessment

FN function analysis was performed through preoperative and postoperative clinical evaluations, with special attention to the MMB. During inpatient care, the MMB was evaluated daily. However, after discharge, it was evaluated at 7 days, 1 month, and according to the clinical follow-up schedule. In cases where in-person evaluation was not possible, the patient was evaluated via telemedicine and was asked to send photographs during the execution of certain facial movements.

In addition, the FN function was classified using the modified House–Brackmann classification system [6]. This is a scoring system (points are awarded for movement of the forehead, mouth, and eyes) that classifies dysfunction from level I to level VI (Table 1).

### 2.4. Surgical Technique

The surgical procedure was timed from the moment of incision to the conclusion of suturing, as documented in the anesthetic record. In both groups, submandibular sialoadenectomy was performed under general anesthesia according to the standard practice procedures. One hour before surgery, patients were administered premedication consisting of midazolam at a dose of 1 to 5 mg. Following the administration of adequate preoxygenation and denitrogenation, the induction of anesthesia was initiated with the intravenous infusion of propofol 2 mg/kg as a bolus (for sedation) in combination with a single reduced dose of rocuronium (0.3 mg/kg), which was not to be repeated during surgery. During the surgical procedure, sedation was maintained using either sevoflurane or desflurane at a Minimal Alveolar Concentration (MAC) ranging from 0.75 to 1.25, or propofol administered via a Target Controlled Infusion (TCI) pump, adjusted to achieve deep sedation, as indicated by a Bispectral Index (BIS) value of approximately 40. Intraoperative analgesia was ensured with a continuous infusion of remifentanil at a rate of 0.05 to 0.25 mg*kg/min. At the end of the surgical procedure, multimodal analgesia was guaranteed to the patient.

The surgical technique involved making a 3 cm incision in the cervical skin, situated 3 cm from the lower edge of the mandible, parallel to it, and approximately 4–5 cm long, along a natural fold of the neck. The incision runs from the anterior border of the sternocleidomastoid muscle to the submental area. The skin and platysma muscle were incised, and the upper and lower flaps were raised to the lower edge of the mandible and below the submandibular gland, respectively [22]. Dissection then proceeded from the superficial cervical fascia to the gland. The facial vessels were identified and ligated/clamped to ensure the protection of the MMB of the FN. In the subjects of Group 2, the mapping or “blind” stimulation of the surrounding tissues of the MMB was performed by using the probe (Medtronic) at no more than two mA without proceeding with the identification of the clamp of the facial vessel. A blunt dissection of the gland was performed from the back to the front, identifying the lingual nerve, Wharton’s duct, and hypoglossal nerve. The Wharton’s duct was then ligated, the gland was excised, and hemostasis was meticulously checked and sutured in layers. In G2, the device employed for IFNM was the Nerve Integrity Monitor (NIM^®^) (Medtronic, Fridley, MN, USA).

In parotid surgery, four recording electrodes were necessary for effective monitoring, whereas in submandibular gland surgery, a single channel was enough to monitor the muscular response of the orbicularis oris, which was innervated by the MMB. After intubation, to ascertain the proper placement of the facial electrode, a tapping test of this muscle was performed, and a response wave in the monitor due to this stimulation was observed.

At the end of the surgical procedure in the patients of Group 2, the system generated a report containing the electromyographic tracings, which were then incorporated into the patient’s medical record. During the postoperative period, patients were closely monitored for potential complications, including deficits of the FN, particularly its MMB. Patients who sustained an FN injury were referred to a rehabilitation program in collaboration with the Physical Medicine and Rehabilitation Department of the “Renato Dulbecco” University Hospital in Catanzaro and subsequently subjected to follow-up checks.

### 2.5. Statistical Analysis

The dataset was subjected to statistical analysis using R software. A descriptive analysis was performed, and univariate and multivariate logistic regression models were used to examine the likelihood of patients developing a postoperative deficit depending on whether or not they underwent IFNM. In addition, univariate and multivariate linear regression models were employed to assess the impact of monitoring on surgical duration. The level of statistical significance was set at *p*-value < 0.05.

## 3. Results

From 1 January 2014 to 31 December 2023, 104 patients underwent submandibular sialoadenectomy at the Maxillofacial Unit of the “Magna Grecia” University of Catanzaro. Of these patients, 101 met the inclusion criteria, while 3 patients were excluded because they had previously undergone surgery in the same location for a different pathology.

Of all the enrolled patients (*n* = 101), 45 (44.56%) were female and 56 (55.44%) were male. Furthermore, 50 subjects (49.50%) were assigned to Group 1, and 51 subjects (50.50%) were assigned to Group 2. Of the 50 G1 patients, 24 (48%) were female and 26 (52%) were male; while of the 51 G2 patients, 21 (41%) were female and 30 (59%) were male.

The mean age was 55 ± 16 years in the entire patient cohort: 56 ± 16 years in G1, and 54 ± 15 years in G2. All patients underwent submandibular sialoadenectomy (100%), of which 50 were in G1 (49.5% of the total patients in G1) and 51 in G2 (50.5%), according to the surgical technique described.

**Figure 1:**
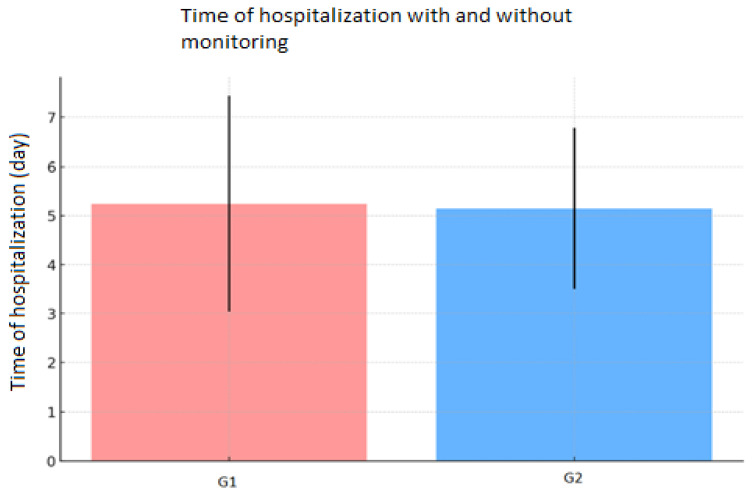
Length of hospital stay: The mean length of hospital stay was similar for both groups, with minimal differences.

The mean length of hospital stay was 5.19 ± 1.93 days for the entire cohort of patients, while it was equal to 5.24 ± 2.20 days for G1 and 5.14 ± 1.64 days for G2 (Figure 1).

**Figure 2:**
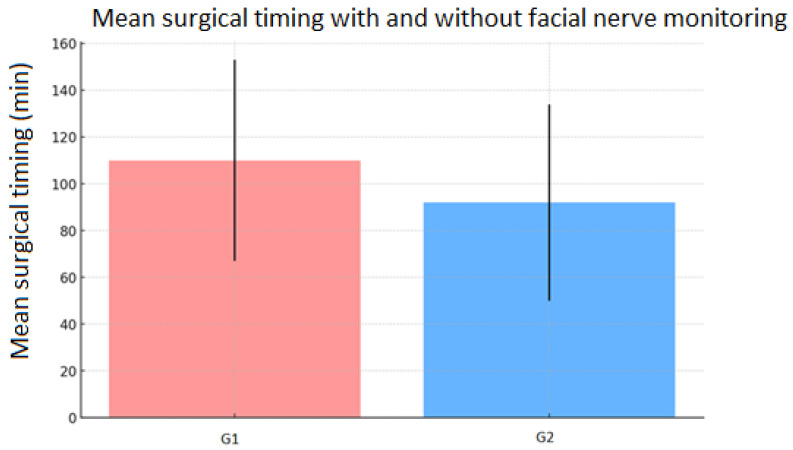
The mean surgical timing in the two groups.

The surgical timing was found to be 99 ± 44 min for the entire cohort of patients: 110 ± 43 min for G1 and 92 ± 42 min for G2 (Figure 2).

**Figure 3:**
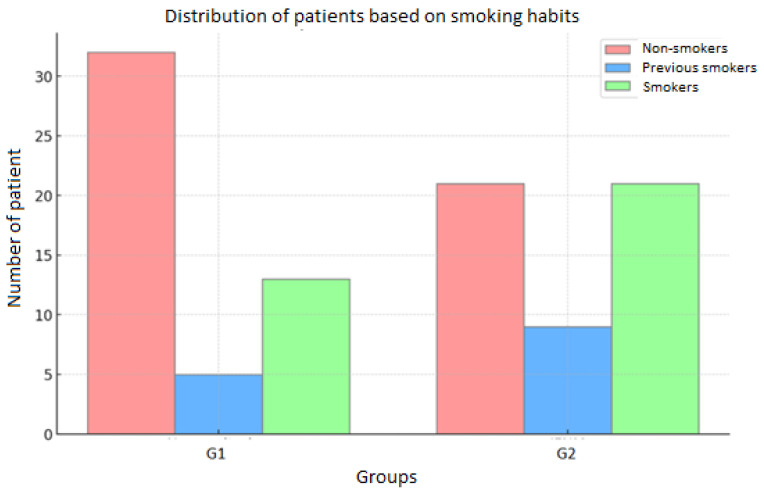
Distribution of patients according to smoking in G1 and G2.

With regard to smoking status, in the entire cohort of patients, 53 (52%) were non-smokers, 14 (14%) were ex-smokers, and 34 (34%) were smokers. In Group 1, 32(64%) patients were non-smokers, 5 (10%) were ex-smokers, and 13 (26%) were smokers. In Group 2, 21 patients (41%) were non-smokers, 9 (18%) were ex-smokers, and 21 (41%) were smokers. (Figure 3).

The diagnosis was classified into three categories: sialolithiasis, benign tumors, and other pathologies (sialadenitis, follicular hyperplasia, Kuttner tumor). A total of 78 patients (77%) underwent surgery for sialolithiasis, with 46 cases classified as G1 (representing 59% of the G1 cohort) and 32 cases classified as G2 (accounting for 41% of the G2 cohort). The patient population who underwent surgical intervention for neoplasms totaled 10 (10%), with 4 (40%) classified as G1 and 6 (60%) as G2. These neoplasms exhibited a size greater than 2 cm, with intraparenchymal localization, and a preoperative cytological diagnosis of pleomorphic adenoma, as confirmed through postoperative histological examination. Patients with other diagnoses numbered 13 (12% of the total patient population), including 5 of G1 (38% of the G1 patients) and 8 of G2 (61% of the G2 patients). (Table 2)

The descriptive analysis of the postoperative paralysis demonstrated that 78 patients (77%) of the entire cohort did not report paralysis, including 38 (76%) of G1 and 40 (78%) of G2.

Conversely, 23 patients (23%) of the entire cohort exhibited varying degrees of paralysis, including 12 patients (24%) of G1 and 11 patients (22%) of G2. Notably, none of the patients reported permanent paralysis (Table 3).

On the day following the surgical procedure, twelve subjects of G1 exhibited varying degrees of paralysis, with four demonstrating grade II dysfunction and eight exhibiting grade III dysfunction. Among the 11 subjects classified as G2, eight exhibited grade II dysfunction, while three demonstrated grade III dysfunction.

Following a period of three months, patients in both groups who initially exhibited grade II dysfunction no longer demonstrated any such dysfunction. Among the patients with grade III dysfunction, eight cases (seven of G1 and one of G2) demonstrated persistent dysfunction after three months. After six months, the dysfunction of grade III persisted in five patients of G1. The detailed data can be found in Table 4.

Postoperatively, patients were monitored for the development of locoregional complications, including edema, wound infection, or hemorrhage, as well as systemic complications, such as dysesthesia or salivary fistula.

The incidence of locoregional postoperative complications was observed to be as follows: edema was noted in thirty subjects (29.7%), with resolution occurring after five to seven days without the need for additional medical therapy. Minor hemorrhages that were not treated were observed in five subjects (4.9%), and wound infection was noted in five subjects (4.9%), with treatment via antibiotics.

A series of statistical investigations were conducted to evaluate the correlation between variables of interest. For all analyses performed, a significance level α of 5% and a confidence interval (CI) of 95% were considered. Initially, univariate and multivariate logistic regression analyses were performed to ascertain the baseline deficit. The odds ratio (OR) for G2 (vs. G1) concerning baseline deficit is 0.87 (*p* = 0.8), indicating that monitoring does not have a significant association with the reduction in risk of postoperative deficit (Table 5).

The second test entailed the implementation of multivariate regression analysis, with the constant comparison of G2 vs. G1, while accounting for confounding variables such as age, sex, smoking status, and vascular ligation. The odds ratio (OR) was determined to be non-significant, with a value of 0.73 and a *p*-value of 0.5 (Table 6).

This OR value indicates that patients in G2 have a 27% lower probability ((0.73–1) x 100) of developing a postoperative deficit compared to those in G1. However, this effect was not statistically significant, as the resulting *p*-value was above the pre-set threshold of 0.05. When the analysis is further adjusted for age (expressed in years), the observed value of 1.01 indicates a 1% increase in the probability of developing a postoperative deficit for each year of age. However, this effect is deemed minimal given the *p*-value of 0.5, which suggests that age does not exert a statistically significant influence. When gender is considered as a factor (female vs. male), an odds ratio of 2.24 indicates that female patients are 2.24 times more likely to develop a postoperative deficit than male patients. This effect did not attain statistical significance, as indicated by a *p*-value of 0.2, which exceeds the 0.05 threshold for statistical significance. A similar outcome was observed when the analysis was repeated for ex-smokers versus non-smokers, with an OR value of 3.64, indicating that ex-smokers are 3.64 times more likely to experience a postoperative deficit compared to non-smokers. While the *p*-value of 0.093 approaches the 0.05 threshold, it does not attain statistical significance. In the smoking vs. non-smoking status analysis, the OR is 1.66, indicating that smokers have a 1.66 times higher probability of developing a post-operative deficit compared to non-smokers. However, this effect did not reach statistical significance (*p*-value = 0.4).

Subsequently, a univariate and multivariate linear regression analysis was performed to examine the relationship between surgical timing and FN monitoring. The results indicated that FN monitoring is significantly associated with a reduction in surgical time. In the univariate regression, G2 exhibited a surgical time reduction of approximately 19 min compared to G1 (*p* = 0.048) (Table 7).

This effect remains statistically significant even in multivariate regression, where the association is even stronger (*p*-value = 0.004), even after adjusting for variables such as age, sex, and smoking status.

Moreover, among patients who smoke, a longer surgical timing was observed compared to patients who do not smoke (always statistically significant with a *p*-value of 0.008), suggesting that smoking status may influence the duration of the intervention (Table 8).

A comparison of G2 and G1 reveals a statistically significant negative correlation between IFNM and surgical timing, with a beta coefficient of −0.28. This indicates an average reduction of 0.28 min in surgical duration. The *p*-value of 0.004 is less than 0.05, which is statistically significant. Conversely, when age (in years) is considered, the Beta is 0.00, indicating that age does not have a significant impact on surgical timing. However, the *p*-value of 0.7 suggests that these data are not statistically significant. When gender is considered as a factor (female vs. male), the Beta coefficient of −0.02 suggests that women have a marginally shorter surgical timing (0.02 min) compared to men. However, this effect is virtually negligible, and it is not statistically significant, as evidenced by the *p*-value of 0.9.

When considering the variable of former smoking status (in comparison to non-smokers), the Beta coefficient increases to 0.09, indicating that ex-smokers experience a slightly longer surgical timing (by 0.09 min). However, this effect is negligible and does not attain statistical significance, as indicated by a *p*-value of 0.5.

Conversely, when considering the smoker status as the independent variable and the non-smoker status as the dependent variable, the Beta value is 0.32. This indicates that smokers have a significantly longer surgical time of 0.32 min compared to non-smokers. These data are statistically significant, as evidenced by the *p*-value of 0.008.

**Figure 4:**
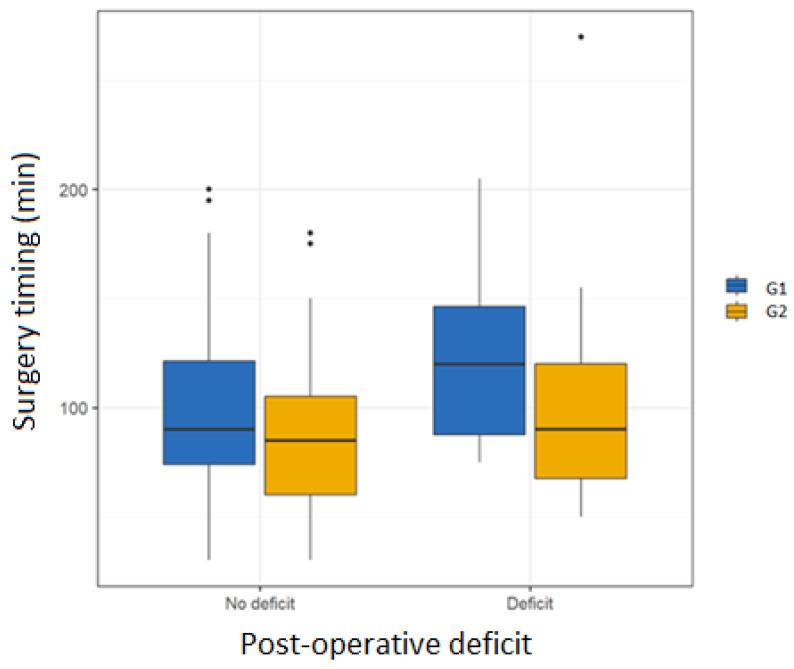
Boxplot showing how surgical timing is significantly reduced in patients undergoing IFNM regardless of postoperative deficit.

The boxplot (Figure 4) is a graphical representation used to visualize the distribution of a dataset and its quartiles. In this graph, the vertical axis represents the surgical timing (expressed in minutes), while the horizontal axis shows the variable post-operative deficit with two categories: “No deficit” and “Deficit”. The data are divided into G1 and G2.

Each boxplot shows the distribution of data for a combination of postoperative deficit and nerve monitoring. The horizontal line within each box represents the median surgical timing.

The edges of the box correspond to the first and third quartiles (the length of the box therefore represents the central 50% values of the data). The vertical lines (whiskers) extend to the minimum and maximum values, excluding outliers (represented as black dots outside the whiskers), i.e., values that deviate significantly from the rest of the data. This graph is useful for comparing surgical times between patients with and without deficits, and for examining the effect of intraoperative monitoring. The graph shows that patients who underwent FN monitoring during surgery had shorter surgical times regardless of postoperative deficit.

**Figure 5:**
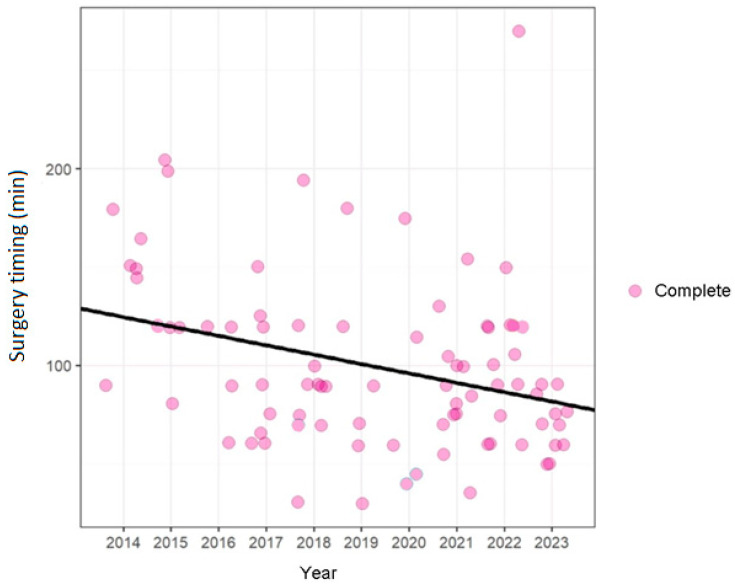
The scatterplot shows how there has been a significant decrease in surgical timing over the years.

The scatterplot (Figure 5) displays the duration of surgery over the period considered (from 2014 to 2023). The vertical axis represents the time of surgery (in minutes), and the horizontal axis shows the year in which the interventions were performed.

Each dot in the graph represents a surgical procedure, with the size and color denoting the completion category. The black trend line, representing linear regression, offers a visual representation of the temporal change in surgical duration. This trend line is instrumental in analyzing trends over time in surgery duration, taking into account the type of surgery. In this particular scatterplot, the points have been slightly shifted to display the entire cohort, given the high number of patients with equal operating time. It is noteworthy that there has been a substantial decrease in surgical time over the years.

## 4. Discussion

The IFNM supports the surgeon in identifying the FN, reporting any accidental manipulation or stimulation, tracing its path, and providing indications on the possible functional outcome of the FN after the operation. It is believed that such monitoring helps to reduce temporary paralysis, promptly warning of potential damage such as stretching or compression, which could compromise the nervous microcirculation [25,26].

Despite the existence of numerous international guidelines and consensus statements addressing the clinical application of IFNM in otology, skull base surgery, and parotid gland surgery, there is a paucity of published standardized protocols on the utilization and interpretation of IFNM in submandibular gland surgery [27,28,29].

The current literature on IFNM in submandibular gland surgery does not provide conclusive evidence demonstrating a direct link between the use of this monitoring and the reduction in the incidence of postoperative deficits, particularly of the MMB [30]. While some studies have suggested that monitoring may be useful in protecting the FN, the available results are not sufficient to establish a statistically significant correlation. However, several investigations have shown that postoperative rates of FN dysfunction in patients undergoing submandibular sialoadenectomy with IFNM may be comparable to those operated without IFNM [19,31,32], as confirmed by the data presented by the authors. In 23 patients with MMB dysfunction (23%), an equal number of patients presented with the damage (12 of G1 and 11 of G2) on the day following surgery. However, a shorter recovery time for the damage was demonstrated.

It is noteworthy that the authors excluded patients with primary or metastatic malignant tumors of the submandibular gland from the study because these tumors can infiltrate the FN’s branches, necessitating a radical surgical strategy.

The study’s univariate and multivariate logistic regression analyses did not demonstrate a statistically significant correlation between IFNM and a substantial reduction in the risk of FN deficiency following submandibular sialoadenectomy (univariate: OR = 0.87, 95% CI from 0.34 to 2.22, *p* = 0.8; multivariate: OR = 0.73, 95% CI from 0.26 to 1.96, *p* = 0.5). In particular, it was observed that in G2, the percentage of patients with post-operative deficit at three months decreased from 22% (11 patients) of the total patients of G2 (51 patients) to 2% (1 patient). In G1, the percentage decreased from 24% (12 patients) to 14% (7 patients). After six months, no patients in G2 exhibited any form of MMB dysfunction, in contrast to the five patients in G1 who continued to manifest grade III dysfunction.

This retrospective study demonstrates that a higher percentage of patients undergoing IFNM (G2) reported a complete resolution of MMB dysfunction in a short time than patients without IFNM (G2). Furthermore, a statistically significant reduction in surgical timing was demonstrated. Univariate and multivariate linear regression analyses revealed that IFNM was associated with a statistically significant reduction in surgical timing, with an average of approximately 19 min less than operations performed without monitoring, as evidenced by univariate linear regression (Beta = −19, 95% CI −37 to −0.16 and *p* = 0.048). This finding suggests a tangible advantage in terms of operative efficiency, potentially mitigating the risk of complications associated with prolonged surgical timing. A statistically significant discrepancy was observed in the surgical timing between smokers and non-smokers, with a *p*-value of 0.008, indicating that smoking status may influence the duration of the intervention. The beta coefficient was −0.32, with a 95% confidence interval of −0.08 to −0.55 and a *p*-value of 0.008, suggesting a potential relationship between smoking and surgical timing.

From the data obtained, the authors suggest that it may be useful during the surgery of the submandibular gland not to proceed with the direct identification of the MMB but to trace its path with the aid of the IFNM. Identifying the facial artery and vein near the lower border of the mandible and ligating these vessels before pulling them cranially to protect the nerve cannot be a standard method because the relationship of the nerve to the facial artery is extremely variable. Of the 11 subjects of G2 in which the facial vessels were not performed and the course of the MMB was identified with IFNM, not only was there a reduction in surgical timing but eight exhibited grade II dysfunction, and three demonstrated grade III dysfunction (versus the 12 subjects of G1 exhibited four demonstrated grade II dysfunction while eight exhibited grade III dysfunction), that resolved in a significantly shorter period of timing than G1 patients. Additionally, MMB identification is often difficult in patients with abundant subcutaneous fat, as many patients have multiple MMBs, and the MMB itself is a thin nerve, so dissection alone may cause paralysis.

Of course, the design of the prospective study will allow us to identify the potential benefits of IFNM in the surgery of benign pathology of the submandibular gland, particularly in reducing the severity of the lesions and shortening the recovery period from transient post-operative paralysis.

## 5. Conclusions

MMB paralysis is a frequent complication of submandibular gland surgery, and IFNM offers surgeons a valuable tool for identifying the MMB during submandibular sialoadenectomy. This retrospective study demonstrated that IFNM is an effective method of reducing the risk of MMB dysfunction. This approach facilitates expedited functional recovery and reduced surgical timing, potentially mitigating the risk of complications associated with extended surgical procedures. Considering the results obtained, we strongly recommend the use of IFNM in surgeries for benign lesions of the submandibular gland. It is imperative to underscore that the employment of monitoring systems should never supersede the expertise, anatomical knowledge, and decision-making ability of the surgeon. While technology can serve as a valid aid, its effectiveness is contingent upon the expertise and judgment of the surgeon, who remains central to the success of the intervention and the well-being of the patient.

## Figures and Tables

**Table 1:** The staging of FN function according to the House–Brackmann classification.

Grading	FN Function
I: Normal	Absence of deficit.
II: Mild dysfunction	Slight facial weakness or other mild dysfunction, normal tone and symmetry at rest; complete closure of the eye without effort; slight asymmetry of the mouth when facial movements occur.
III: Moderate dysfunction	No facial weakness with synkinesis and complete eye closure and good forehead movement with effort.
IV: Moderate–severe dysfunction	Obvious facial weakness. Incomplete eye closure, no forehead movement, asymmetrical mouth movement, and synkinesis.
V: Severe dysfunction	Little to no ability to smile, frown, or make other facial expressions. The closure of the eye is incomplete, and there is no forehead movement.
VI: Complete paralysis	No facial motion.

**Table 2:** Characteristics of the patient cohort of G1 and G2.

Characteristics	Total Number = 101	G1 N = 50	G2 N = 51
Age (years)	55 ± 16	56 ± 16	54 ± 15
Sex (female)	45 (45%)	24 (48%)	21 (41%)
Hospitalization (days)	5,19 ± 1,93	5,24 ± 2,20	5,14 ± 1,64
Surgical timing (minutes)	99 ± 44	110 ± 43	92 ± 42
**Smokers**			
No smokers	53 (52%)	32 (64%)	21 (41%)
Ex-smokers	14 (14%)	5 (10%)	9 (18%)
Smokers	34 (34%)	13 (26%)	21 (41%)
**Diagnosis**			
Sialolithiasis	78 (77%)	46 (59%)	32 (41%)
Benign tumor (pleomorphic adenoma)	10 (10%)	4 (40%)	6 (60%)
Other (sialadenitis, follicular hyperplasia, Kuttner tumor)	13 (12%)	5 (38%)	8 (61%)

**Table 3:** Postoperative paralysis rates in the two groups.

Characteristics	Total Number = 101	G1 N = 50	G2 N = 51
**Post-surgery deficit**			
No deficit	78 (77%)	38 (76%)	40 (78%)
Deficit day one	23 (23%)	12 (24%)	11 (22%)
**Deficit after three months**			
No deficit	92 (92%)	43 (86%)	50 (98%)
Deficit	8 (8%)	7 (14%)	1 (2%)
**Deficit after six months**			
No deficit	96 (95%)	45 (90%)	51(100%)
Deficit	5 (5%)	5 (10%)	

**Table 4:** FNI assessed by House–Brackmann classification.

DAY	G1	No Facial Paralysis	G2	No Facial Paralysis
Day 1	Gr II: 4/12 Gr III: 8/12	38	Gr II: 8/11 Gr III: 3/11	40
Days 90	(Gr III): 7/12	43	Gr III: 1/11	50
Days 180	(Gr III): 5/12	45		51

**Table 5:** Univariate logistic regression for the association between baseline deficit and IFNM in G2 vs. G1.

Characteristic	OR	95% CI	*p*-Value
G2 (vs. G1)	0.87	0.34, 2.22	0.8

**Table 6:** Multivariate logistic regression for the association between baseline deficit and G2 (vs. G1) adjusted for age, sex, and smoking status.

Characteristic	OR	95% CI	*p*-Value
G2 (vs. G1)	0.73	0.26, 1.96	0.5
Age (years)	1.01	0.98, 1.05	0.5
Females (vs. Males)	2.24	0.73, 7.41	0.2
No smokers			
Ex-smokers	3.64	0.79, 17.0	0.093
Smokers	1.66	0.44, 6.26	0.4

**Table 7:** Univariate linear regression for the association between surgical timing and IFNM (G2 vs. G1).

Characteristic	Beta	95% CI	*p*-Value
G2 (vs. G1)	−19	−37, −0.16	0.048

**Table 8:** Multivariate linear regression for the association between surgical timing and G2 (vs. G1) adjusted for age, sex, smoking status.

Characteristic	Beta	95% CI	*p*-Value
G2 (vs. G1)	−0.28	−0.46, −0.09	0.004
Age	0.00	0.00, 0.01	0.7
Females (vs. Males)	−0.02	−0.23, 0.19	0.9
No smokers			
Ex-smokers	0.09	−0.20, 0.39	0.5
Smokers	0.32	0.08, 0.55	0.008

## Data Availability

The data are available on request from the corresponding author.
